# Efficacy of local Dexamethasone used in Operation Area after Lumbar Microdiscectomy on postoperative infection

**DOI:** 10.12669/pjms.41.10.11756

**Published:** 2025-10

**Authors:** Murat Yucel, Eyup Cetin, Ozkan Arabaci, Mehmet Edip Akyol

**Affiliations:** 1 Murat Yucel, Faculty of Medicine, Department of Neurosurgery, Yalova University, Yalova, Turkey; 2 Eyup Cetin, Department of Neurosurgery, Haydarpasa Numune Training, Research Hospital, Istanbul, Turkey. Faculty of Medicine, Department of Neurosurgery, Van Yuzuncu Yil University, Turkey; 3 Ozkan Arabaci, Faculty of Medicine, Department of Neurosurgery, Van Yuzuncu Yil University, Turkey; 4 Mehmet Edip Akyol, Faculty of Medicine, Department of Neurosurgery, Van Yuzuncu Yil University, Turkey

**Keywords:** Dexamethasone, Deep infection, Local infection, Microdiscectomy, Postoperative infection

## Abstract

**Background & Objective:**

Postoperative wound infection, although relatively uncommon, remains a significant complication of spinal surgery. Preventive strategies must therefore be rigorously implemented before, during and after surgery. While local steroid administration is frequently employed during lumbar microdiscectomy to reduce neural edema, limited evidence exists regarding its effects on infection rates. This study aimed to evaluate the impact of locally applied dexamethasone in the surgical site on the incidence of postoperative infection.

**Methodology:**

This retrospective observational study was conducted between January 2020 to December 2022 at the Neurosurgery Departments of Van Yüzüncü Yıl University Medical School. A total of 200 patients (89 females, 111 males) who underwent lumbar microdiscectomy were included. Patients were divided into two groups: those who received local dexamethasone (8 mg) applied to the surgical site (n = 54) and those who did not (n = 146). No systemic steroids were used in either group.

**Results:**

In the dexamethasone group, no patients developed superficial wound infection, whereas 11 patients in the non-dexamethasone group did. No deep infection was observed in either group. Although the reduction in infection was not statistically significant, the local infection rate was significantly higher in the non-dexamethasone group (p < 0.05).

**Conclusion:**

Local administration of dexamethasone following lumbar microdiscectomy was associated with a lower rate of superficial wound infection compared with no dexamethasone use. Postoperative infection remains an important clinical concern in spinal surgery, and careful attention to perioperative preventive measures is warranted.

## INTRODUCTION

Low back pain and radicular pain are among the most common causes of medical and socioeconomic burden, with approximately 80% of the adult population experiencing low back pain at least once in their lifetime.^1^ A conservative approach is generally the first-line treatment for Lumbar Disc Herniation (LDH). Surgical intervention is considered in cases where symptoms persist despite conservative treatment, when a progressive motor deficit develops, or in the presence of Cauda Equina syndrome, caused by the compression of the nerve roots. The most common surgical procedure is the traditional lumbar microdiscectomy.

Although lumbar microdiscectomy is widely regarded as a safe and effective procedure, postoperative complications can occur. These include superficial and deep wound infections, dural tears, recurrence of disc herniation, and postoperative motor deficits.^2^ Among these, infection is the most important early complication after spinal surgery. The reported incidence of postoperative wound infection varies between 0.7% and 11.6%.^3^ Several risk factors have been identified, including advanced age, long-term use of systemic steroids, diabetes mellitus (DM), intravenous drug usage, organ transplantation, protracted operations, significant blood loss, malnutrition and instrumentation.^4^

Local administration of corticosteroids is often employed during microdiscectomy to reduce neural edema and postoperative pain. However, concerns remain regarding their potential impact on wound healing and infection risk. To address this, we conducted a retrospective study to evaluate the effect of locally applied dexamethasone on postoperative infection rates in patients undergoing lumbar microdiscectomy.

## METHODOLOGY

This retrospective observational study reviewed the clinical and laboratory records of 200 patients (89 females, 111 males) who underwent single-level lumbar microdiscectomy from January 2020 to December 2022 at the Neurosurgery Departments of Van Yüzüncü Yıl University Medical School. Following surgery, patients were assigned to one of two groups: the dexamethasone group (n = 54), in which a single ampoule of dexamethasone (8 mg) was applied locally to the surgical site, or the control group (n = 146), which received no local medication.

### Ethical Approval:

This study was approved by the Van Yüzüncü Yıl University Non-Invasive Clinical Research Ethics Committee (approval no. 2022/10-28; October 14, 2022). As this investigation involved retrospective analysis of existing patient records, the requirement for informed consent was waived in accordance with national regulations.

Patients were eligible for inclusion if they were aged 18-75 years, had magnetic resonance imaging-confirmed single-level lumbar disc herniation and underwent primary lumbar microdiscectomy during the specified period. Exclusion criteria comprised systemic steroid use within three months prior to surgery, active infection at any body site at the time of surgery, history of previous lumbar spine surgery and immunocompromised status (for example, HIV infection or ongoing chemotherapy).

Demographic and clinical data collected included age, sex, timing of infection diagnosis, presence of postoperative fever and wound-swab culture results. In addition, laboratory parameters-white blood cell count (WBC), erythrocyte sedimentation rate (ESR) and C-reactive protein (CRP)-were recorded preoperatively and monitored at regular intervals postoperatively.

### Statistical analysis:

All statistical analyses were performed using SPSS 28.0 for Windows (IBM Corp., Armonk, NY). Continuous variables are presented as mean ± standard deviation or median (range) and were compared using the Mann-Whitney U-test. Categorical variables were analyzed with the chi-square test or Fisher’s exact test, as appropriate. A p-value of less than 0.05 was considered to indicate statistical significance.

## RESULTS

The proportion of female patients was higher in the dexamethasone group compared with the non-dexamethasone group (61.1% vs. 38.4%) even though there was no discernible difference in the two groups’ mean ages (p>0.05). There was no noticeable difference in the rate of dural injury between the two groups (p>0.05) (Table-I).

**Table-I:** Demographic and Clinical Characteristics of Patients Undergoing Lumbar Microdiscectomy With and Without Local Dexamethasone Application.

		Non-Dexamethasone				Dexamethasone				p	
		Mean±SD/n-%			Median	Mean±SD/n-%			Median
Age (years)		46.5	±	13.4	45,0	45.1	±	12.6	43.5	0.380	^m^
Gender	Female	56		38.4%		33		61.1%		***0.004***	^X²^
	Male	90		61.6%		21		38.9%			
Dural Injury	No	141		96.6%		49		90.7%		0.093	^X²^
	Yes	5		3.4%		5		9.3%			
Systemic Diseases	No	135		92.5%		47		87.0%		0.234	^X²^
	Yes	11		7.5%		7		13.0%		
*HT*		7		63.6%		1		14.3%		0.066	^X²^
*DM*		4		36.4%		4		57.1%		0.630	^X²^
*Hypothyroidism*		0		0.0%		2		28.6%		0.137	^X²^
*Asthma*		1		9.1%		0		0.0%		1.000	^X²^
Local Infection	No	135		92.5%		54		100.0%		***0.038***	^X²^
	Yes	11		7.5%		0		0.0%			
Surgical Side	Right	54		37.0%		13		24.1%		0.086	^X²^
	Left	92		63.0%		41		75.9%		
Duration of Surgery(minutes)		95.8	±	30.5	90,0	124.9	±	44.8	120,0	***0.000***	^m^
Surgical Site	L2-3	4		2.7%		1		1.9%		1.000	^X²^
	L3-4	3		2.1%		0		0.0%		0.565	^X²^
	L4-5	90		61.6%		33		61.1%		0.945	^X²^
	L5-S1	49		33.6%		20		37.0%		0.646	^X²^
Disc Herniation Recurrent	No	133		91.1%		46		85.2%		0.226	^X²^
	Yes	13		8.9%		8		14.8%		
Hospital Stay (days)		1.6	±	1.8	1.0	2.3	±	1.5	2.0	***0.000***	^m^

m Mann-Whitney U-test /

X² Chi-square test (Fisher test)

HT: Hypertension, DM: Diabetes Mellitus, SD: Standard Deviation.

The prevalence of systemic disorders did not differ significantly between the two groups, including hypertension (HT), DM, hypothyroidism and asthma (*p*>0.05 for all the systemic disorders). However, the non-dexamethasone group’s local infection rate was substantially greater than the dexamethasone group (*p*<0.05) (Table-I).

There was no discernible difference between the two groups in terms of the injured side (right *vs*. left) (*p*>0.05). Duration of surgery was obviously shorter in the non-dexamethasone group than in the dexamethasone group (*p*<0.05) (Table-I).Similarly, there was no discernible difference between the two groups with regard to the surgical location or the recurrence rate (p>0.05 for both groups). However, the non-dexamethasone group’s length of hospitalization was much shorter than the dexamethasone group (*p*<0.05) (Table-I).

## DISCUSSION

**Fig.1:**
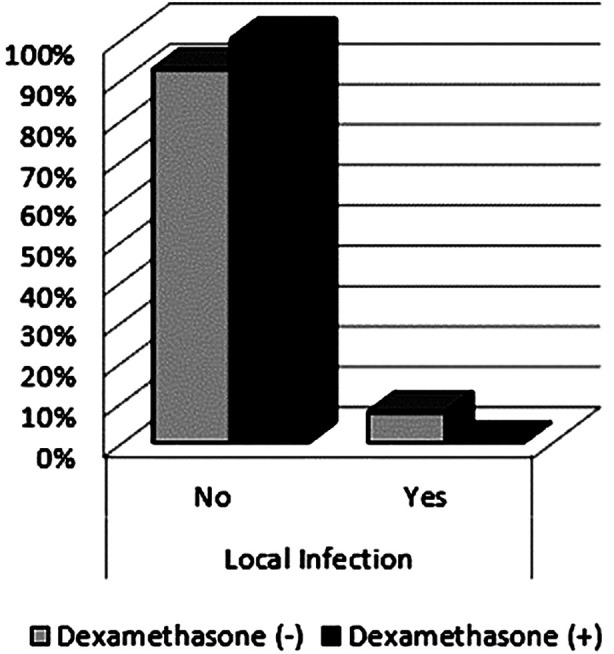
Comparison of local infection rates between the Dexamethasone and Non-Dexamethasone Groups.

In this retrospective cohort of 200 patients undergoing single-level lumbar microdiscectomy, the topical administration of 8-mg dexamethasone to the surgical site was associated with a 0% rate of superficial wound infection, in contrast to a 7.5% infection rate in the control group (*p* < 0.05; Fig.1). No deep infections were observed in either cohort. These findings suggest that local application of dexamethasone may significantly reduce the incidence of superficial postoperative infections without increasing the risk of deep or systemic infectious complications, even in the presence of known perioperative risk factors.

The observed protective effect is biologically plausible and consistent with the pharmacological properties of corticosteroids. Glucocorticoids such as dexamethasone inhibit the activity of phospholipase A_2_, thereby suppressing prostaglandin synthesis and reducing local tissue edema.^5^ Attenuation of inflammation may enhance local microcirculation and facilitate the delivery of antimicrobial agents to the surgical field. Additionally, corticosteroids contribute to the stabilization of neural membranes and modulation of peripheral nociceptor input, potentially improving tissue tolerance to surgical trauma.^6^,^7^ Compared to methylprednisolone, dexamethasone has a shorter half-life and lower lipophilicity, which may reduce the risk of prolonged immunosuppression at the application site.^8^

Despite these theoretical benefits, prior studies have expressed concern regarding the potential for corticosteroids to increase the risk of postoperative infections. Lowell et al. reported three cases of epidural abscess in a series of 31 patients who received intraoperative methylprednisolone, suggesting a causal association with steroid-induced immunosuppression in the epidural space.^9^ Similarly, Akinduro et al. noted a nonsignificant trend toward higher infection rates following intraoperative epidural steroid use in their systematic review.^10^ Rich also observed an increased incidence of postoperative infection in a retrospective analysis of patients who received steroids following discectomy.^11^ These discrepancies may be attributable to variations in steroid type, dosage and route of administration. In contrast to the epidural delivery of high-dose methylprednisolone, the localized paraspinal application of a single 8 mg dexamethasone dose in the present study may exert anti-inflammatory effects limited to superficial tissues, thereby minimizing systemic immunosuppression.

The superficial wound infection rate in our control group (7.5%) exceeded those reported in several large-scale European and North American studies, which typically range from 0.7% to 2% for minimally invasive spinal procedures.^12^ In the Turkish context, Blam et al. documented a 3.7% infection rate for elective spine surgeries and a 9.4% rate for trauma cases, likely reflecting greater surgical complexity and patient comorbidities.^13^ Çetin and Yücel reported that the topical application of rifampicin reduced postoperative infection rates from 8.9% to 2.2% in patients undergoing lumbar microdiscectomy.^14^ The relatively high infection rate observed in our control group (Fig.1) may be attributable to variability in perioperative antibiotic protocols, inter-institutional differences in surgical technique across the three centers and the inclusion of patients with underlying conditions such as diabetes mellitus and hypertension.

Numerous intraoperative and patient-related factors have been implicated in the development of postoperative infections, including the duration of surgery, incision length, retraction intensity and duration, cerebrospinal fluid leakage, instrumentation use and nutritional status.^15^ Notably, although operative time was longer in the dexamethasone group, no superficial infections were recorded in this cohort (Fig.1). This suggests that local anti-inflammatory modulation by dexamethasone may counteract infection risks associated with prolonged surgical exposure.

Postoperative infections may manifest in the immediate postoperative period or present later as chronic diskitis if not promptly diagnosed and managed.^16^ Standard treatment involves targeted antibiotic therapy based on wound culture results. In cases with negative cultures, empirical broad-spectrum antibiotics are typically administered for 6-8 weeks, whereas pathogen-specific regimens are used when cultures are positive.^17^ Strategies such as local corticosteroid application that reduce superficial infection rates may contribute to decreased antibiotic utilization and help mitigate the emergence of antimicrobial resistance.

### Strengths and Implications:

To our knowledge, it is among the larger single-center studies specifically evaluating the effect of local dexamethasone on postoperative infection after lumbar microdiscectomy. The inclusion of 200 patients provides a robust sample size for this type of surgical research. Clearly defined inclusion and exclusion criteria further enhance the reliability of the findings. Importantly, both study groups underwent the same surgical procedure under standardized perioperative conditions, which supports comparability. Collectively, these factors suggest that local dexamethasone may represent a simple, low-cost, and clinically relevant adjunct to reduce superficial wound infections in lumbar spine surgery.

### Limitations:

Several limitations should also be considered. First, the retrospective design restricts causal inference and carries the risk of selection bias. Second, although all patients underwent the same standardized operation, the procedures were performed by different surgeons, which could introduce minor variations in surgical technique and intraoperative management. Third, as the study was conducted at a single institution, the findings may not be fully generalizable to other clinical settings. Finally, the absence of long-term follow-up data limited the ability to assess recurrence or delayed postoperative infections.

## CONCLUSION

In this study, the local application of dexamethasone during lumbar microdiscectomy was associated with a lower rate of superficial wound infection compared with surgery without dexamethasone. These findings suggest that locally applied dexamethasone may be a useful adjunct to enhance postoperative safety in spinal surgery. Postoperative infection remains an important clinical challenge, and careful adherence to perioperative preventive measures is essential.

### Future directions:

Future studies should aim to confirm these findings in prospective, multicenter, randomized controlled trials, which would help reduce institutional and surgeon-related variability. Long-term follow-up would be valuable to assess whether local dexamethasone use influences recurrence rates, functional recovery, or late-onset complications. In addition, mechanistic studies examining the local immunological and microbiological effects of dexamethasone could provide further insight into its potential protective role in postoperative infection prevention.

## Data availability:

The datasets used and/or analyzed during the current study available from the corresponding author on reasonable request.

### Author’s Contributions

**MY and EC:** were responsible for the conceptualization of the study.

**MY and OA:** contributed to the methodology and data collection.

**MEA and OA:** Performed data curation, while **EC** and **MEA** conducted the formal analysis. The investigation was carried out by **MY** and **OA**.

The original draft of the manuscript was written by **MY** and it was reviewed and edited by **EC** and **MEA**.

**EC** supervised the overall study process.

All authors have read and approved the final version of the manuscript.

**MY:** Assumes full responsibility for the accuracy and integrity of the data and the content presented in this article.
